# Marine-Derived Defenses Against HIV: Emerging Bioactive Molecules from the Seas

**DOI:** 10.3390/md24020070

**Published:** 2026-02-07

**Authors:** Tiago Santos, Ana Pintão, Carolina S. Marques, Pedro Brandão

**Affiliations:** 1 Egas Moniz Center for Interdisciplinary Research (CiiEM), Egas Moniz School of Health and Science, 2829-511 Caparica, Portugal; tsantos@egasmoniz.edu.pt (T.S.); apintao@egasmoniz.edu.pt (A.P.); 2 Department of Chemistry and Biochemistry, Faculty of Sciences, University of Lisboa, Campo Grande, 1749-016 Lisbon, Portugal; csimarques@ciencias.ulisboa.pt; 3 iBB-Institute for Bioengineering and Biosciences, Department of Bioengineering, Instituto Superior Técnico, University of Lisboa, 1049-001 Lisbon, Portugal; 4 Associate Laboratory i4HB–Institute for Health and Bio-Economy, Instituto Superior Técnico, University of Lisboa, Av. Rovisco Pais, 1049-001 Lisbon, Portugal; 5 CQC-IMS, Department of Chemistry, University of Coimbra, Rua Larga, 3004-535 Coimbra, Portugal

**Keywords:** cnidarian, sponge, algae, tunicate, HIV, antiretroviral

## Abstract

Marine ecosystems have yielded a remarkable diversity of bioactive metabolites with relevance for antiviral drug discovery. This article reviews recent advances in marine-derived compounds investigated as anti-HIV agents. Metabolites, such as sulfated polysaccharides, lectins, alkaloids, and terpenoids, display inhibitory activity across multiple stages of the HIV life cycle, including viral entry, reverse transcription, integration, and maturation. From sponge-inspired development of AZT to the application of Griffithin in clinical trials for the prophylaxis of the HIV infection, recent discoveries showcase the chemical diversity of marine ecosystems and validate their utility as hit and compound sources in drug discovery. We highlight possible mechanisms of action, as well as translational hurdles from research to clinical trials. Overall, marine biodiversity represents a valuable and underexploited reservoir for the development of novel HIV therapeutics.

## 1. Introduction

Marine ecosystems host a wide plethora of structurally novel bioactive natural products. The potency, new mechanisms of action, and efficacy of many of these compounds have attracted the interest of several research groups, which have focused their attention on the identification and development of new drug leads for many therapeutic areas [1,2]. Oceans house many organisms, including invertebrates, such as sponges [3,4,5], cnidarians [6,7,8], and algae [9,10,11,12] capable of generating unique scaffolds that do not exist or are seldom found in terrestrial sources, enabling access to new chemical space for the discovery of new drug candidates [13]. Currently, there are 14 marine compound-derived drugs available on the market, and about 23 natural products in Phase I to Phase III clinical trials [14].

**Figure 1:**
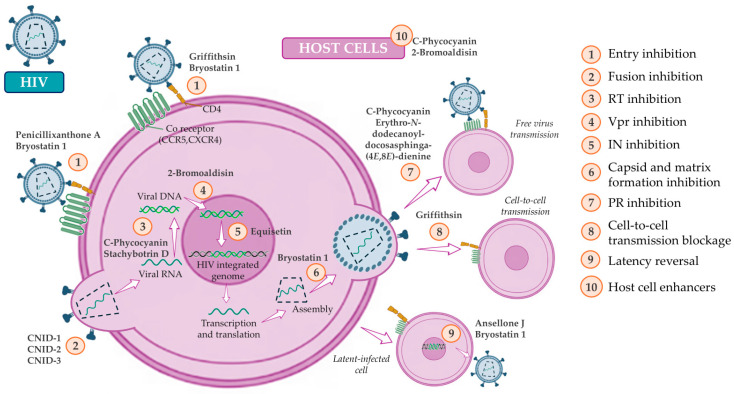
Overview of anti-HIV modes of action from marine bioactive compounds. Numbers represent the mechanisms of action (RT, HIV reverse transcriptase; IN, HIV integrase; PR, HIV protease). Created in BioRender. Nogueira, P. (2025) https://BioRender.com (accessed on 17 January 2026).

Several marine-derived compounds have been tested for their antiviral activity, in particular against human immunodeficiency virus (HIV), the infectious agent responsible for the acquired immunodeficiency syndrome (AIDS) over the past decades [15,16,17]. The current anti-HIV therapeutic options have improved disease control and patients’ quality of life, but they still face persistent challenges. As patients live longer and the disease shifts to chronic management, lifelong therapy for people living with HIV increases the probability of cumulative toxicity of these drugs. Furthermore, associated costs will tend to increase, whereas therapeutic compliance will remain highly dependent on user-friendly posology, which might hinder the therapeutic success in low-income countries often facing high rates of infection, making it a major global public health concern [18,19]. Acquired and transmitted HIV drug resistance is also a major drawback for therapeutic success, and, as the drug resistance rates continue to increase, the need for more efficient, safer, and less prone to resistance drug candidates is an emergent challenge in drug discovery [20,21,22]. Marine natural products display diverse mechanisms of action (Figure 1), ranging from direct virucidal activity to entry inhibitors and immune-modulatory activity, and therefore can operate as new therapeutic options or as co-adjuvants to existing therapeutic regimens [23,24,25]. In recent years, there has been a clear growth in the prospecting of marine compounds with HIV activity, especially from algae, marine fungi, and dinoflagellates. Most of the findings are still in the in vitro or in silico phase, but they already reveal new structural classes and multiple targets in the viral cycle.

Riccio and co-workers summarized the landscape of the research in this field between 2010 and 2020 [26]. In this work, we present an overview of the literature between 2020 and 2025 on the most promising marine bioactive compounds isolated from sponges, tunicates, cnidarians, algae, and marine microorganisms; symbiosis established between marine species; and research updates on the mechanism of action of marine products targeting HIV. These molecules might present a developmental opportunity for new drug candidates for the prevention or therapy of HIV infections.

## 2. Sponges as Sources of Anti-HIV Compounds

Sea sponges are marine invertebrates of the animal phylum *Porifera*. Considered some of the most ancient, diverse, and ecologically significant metazoans in the ocean, they are valuable sources of bioactive compounds on their own or through symbiotic relationships established with multiple microorganisms [27,28].

**Figure 2:**
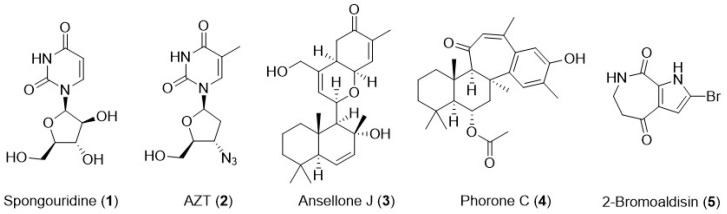
Examples of marine natural products (**1**, **3**–**5**) isolated from sponges and a nature-inspired drug (**2**) with relevant anti-HIV activity.

The interest in sponges as sources of valuable bioactive scaffolds increased in the middle of the last century, with the discovery of arabinosyl glycosides from the marine sponge *Tectitethya crypta* [29]. Among these compounds, spongouridine (**1**, Figure 2) stood out for its ability to mimic natural nucleosides despite its unusual arabinose sugar moiety, enabling viral DNA polymerase inhibition [30]. These findings prompted researchers to search for synthetic and semi-synthetic derivatives with promising pharmacological activity, including AZT (**2**, Figure 2), or zidovudine, the first drug approved against HIV infection, a reverse transcriptase inhibitor. Other drugs with the same mechanism of action, such as lamivudine and abacavir, were also developed and still play a crucial role in combination therapy against HIV.

Recently, Wang et al. isolated new sesterterpenoids from a *Phorbas* sp. and evaluated their bioactivity. Among the five new compounds, ansellone J (**3**, Figure 2) and phorone C (**4**, Figure 2) exhibited effective and potent in vitro HIV-1 latency reversal activity [31]. Latency reversal agents (LRAs) aim at the eradication of the viral reservoir. HIV-1 latently infected cells do not actively produce the virus but are able to resume viral production, in particular when antiretroviral therapy is absent. These agents, so far, have proven to be capable of reducing the viral reservoir but have not displayed efficacy in fully eradicating it [32]. When evaluated in vitro in the Jurkat helper cell line (J-Lat 9.2), which reports latent HIV reactivation through GFP expression, ansellone J surpassed the activity of prostratin, a well-established protein kinase C (PKC) activator used as a benchmark LRA. GFP expression in prostratin at 10 and 30 µM was 11.1 ± 4.0% and 11.7 ± 5.3% of positive events, respectively. Ansellone J at 3 µM showed 27.3 ± 3.4% GFP expression, indicating improved LRA activity. Inhibition experiments using the PKC blocker Gö 6983 confirmed that ansellone J latency-reversing activity acts through PKC-dependent signaling. To further confirm the latency reversal activity, ansellone J was evaluated in a primary cell context using CD4^+^ T cells isolated from HIV antiretroviral-suppressed donors, and its effects were comparable to prostratin. Ansellone J induced HIV latency reversal at a tenfold lower concentration, making it a promising candidate for the development of an LRA-based clinical trial to explore a “shock and kill” approach to achieving the viral reservoir eradication and, therefore, a cure for HIV-1 infections.

Morita and co-workers evaluated a series of pyrrolactams isolated from *Stylissa massa* against another potential therapeutic target, the viral protein R (Vpr) [33]. Vpr is a small, conserved protein (14 kDa) present in HIV-1 that modifies host cell metabolism and oxidative status, leading to a multifactorial mechanism of action that renders it a promising therapeutic target [34]. Among the isolated compounds, brominated pyrrolactam 2-bromoalsidin (**5**, Figure 2) proved to be effective against TREx-HeLa-Vpr at a concentration of 10 μM, with potency comparable to that exhibited by damnacanthal (positive control). This new natural product might open the door to new drug candidates targeting Vpr.

These recent findings showcase how sponges can offer valuable scaffolds for new drug candidates to address HIV infection, adding to other previously reported marine natural products such as mirabamides from *Siliquariaspongia mirabilis* (HIV-1 fusion and entry inhibitors) [35], neamphamide A from *Neamphius huxleyi* [36], and callipeltin A from *Callipelta* sp. [37], and bromotyrosine derivatives from *Verongula rigida* and *Aiolochoria crassa* [38]. These discoveries highlight the chemical diversity and therapeutic potential of marine sponges in the search for novel anti-retroviral agents.

## 3. Tunicates as Sources of Anti-HIV Compounds

Another group of marine invertebrates is ascidians, which include tunicates and sea squirts. There are approximately 3000 recognized species of tunicates worldwide, and they have been reported to possess several pharmacologically active compounds [39,40].

**Figure 3:**
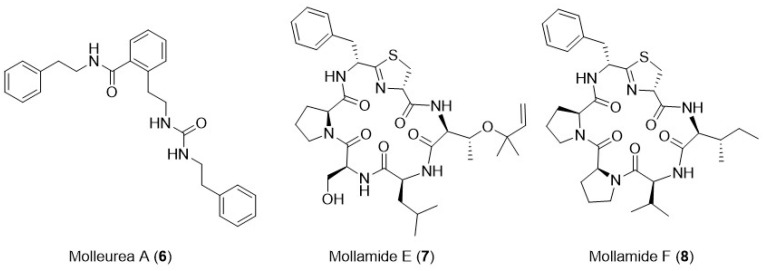
Examples of marine natural products isolated from tunicates with relevant anti-HIV activity.

*Didemnum molle* displays several compounds with promising anti-HIV activity, namely tris-phenethyl urea (molleurea A, **6**, Figure 3), thiazoline peptide, mollamides E and F (**7** and **8**, respectively, Figure 3) [41]. Among these compounds, mollamide F presented HIV integrase inhibition activity (39 μM) and, in a cytoprotective cell-based assay, showed an IC_50_ of 78 μM. Molleurea A exhibited an IC_50_ of 60 μM in the same cytoprotective cell-based assay [41].

## 4. Cnidarians as Sources of Anti-HIV Compounds

The phylum Cnidaria is a very heterogeneous group with thousands of species, which includes corals, sea anemones, box jellyfish, siphonophores, medusae, hydroids, and true jellyfish, among others. This highly diverse group of marine invertebrates is a source of a plethora of bioactive compounds, namely terpenoids, steroids, and eicosanoids. From an evolutionary perspective, these species do not possess a traditional protection system and live in habitats populated by numerous viruses, bacteria, and parasites. Nevertheless, they endure these habitats, increasing researchers’ interest in better understanding their defense mechanisms against pathogens [42,43].

**Figure 4:**
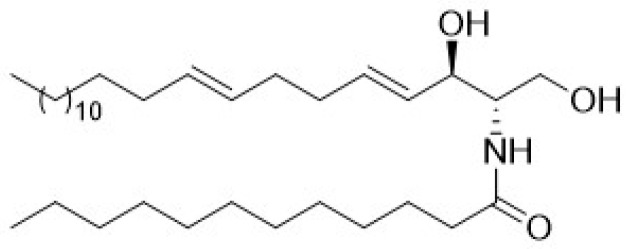
Examples of a marine natural product isolated from *Litophyton arboreum* (Cnidaria) with relevant anti-HIV activity.

The interest in studying cnidarians as sources of antiviral drug candidates, and in particular targeting HIV, emerged at the turn of the century, with the report of cembranoid diterpenes from soft coral *Lobophytum* sp. with high activity (EC_50_ approximately 3–5 µg/mL) in a cell-based in vitro anti-HIV assay [44]. Subsequently, researchers reported anti-HIV activity, through the inhibition of HIV-1 protease (HIV-1 PR), exhibited by the lipophilic fraction of the jellyfish *Cassiopea andromeda* [45]. The same group reported that erythro-*N*-dodecanoyl-docosasphinga-(4*E*,8*E*)-dienine (**9**, Figure 4), isolated from the soft coral *Litophyton arboreum*, showed potent HIV-1 PR inhibitory activity in the absence of cytotoxicity against two cell lines tested. In silico docking of most compounds isolated from *Litophyton arboreum* presented comparable scores to that of acetyl pepstatin, a known HIV-1 PR inhibitor [46].

O’Keefe et al. registered a USA patent on antiviral cnidarins (CNID-1, CNID-2, and CNID-3), proteins isolated from the aqueous extract of the soft coral *Synthecium* sp., which exhibit potent activity against HIV-1 (at low nano- to picomolar concentrations). These compounds have been shown to inhibit HIV viral fusion but not attachment, indicating a post-CD4 association target is likely responsible for their activity [47].

Another point of interest is cnidarian toxins. Their potential is not adequately investigated for bioactive properties, except for neurotoxins, which are often evaluated for nervous system pathologies. The screening of these toxins for other bioactivities through different in vitro, ex vivo, and in vivo models might lead to new drug candidates [48].

## 5. Algae as Sources of Anti-HIV Compounds

Algae diversity can be clearly stated by the fact that they were recently included in four kingdoms (*Eubacteria*, *Chromista*, *Plantae*, and *Protozoa*), fourteen phyla, and sixty-three classes [49]. Algae of marine origin display diverse structural complexity, ranging from unicellular microorganisms, such as cyanobacteria, diatoms, and dinoflagellates (microalgae) to multicellular forms (macroalgae or seaweeds), consisting of brown algae (*Phaeophyta*), red algae (*Rhodophyta*), and green algae (*Chlorophyta*) [50].

These marine organisms have adapted to survive and thrive in intricate and competitive ecosystems, in various conditions, including nutrient-poor habitats, low-light exposure, and temperature variations, all of which can impact their biochemical composition, with the development of specialized secondary metabolites as adaptive tools and defense mechanisms [11,51]. As a result, researchers identified a myriad of algae-derived compounds exhibiting several bioactivities, from anti-inflammatory and anticancer to antiviral, including antiretroviral bioactive molecules [9,52,53].

### 5.1. Macromolecules—Polysaccharides and Proteins

Amongst the most in-depth studied marine algae anti-HIV compounds are sulfated polysaccharides, such as fucoidans from brown algae [54,55], carrageenans from red algae [56,57], and rhamnan sulfates from green algae [58]. Their mechanisms of action include viral entry blockage, fusion inhibition, reverse transcriptase inhibition, and prevention of cell-to-cell fusion and viral spread [59]. Some polyphenols, such as phlorotannins, and diterpenes, such as dolabelladienetriol, also exhibit preclinical activity against HIV [60,61,62].

Griffithin (GRFT) is a lectin protein isolated from the red algae *Griffithsia* sp., which displays notable activity against HIV-1 subtypes A, B, and C, with an EC_50_ of just 40 pM [63]. GRFT acts as an entry inhibitor by binding high-mannose glycan structures on the HIV envelope glycoprotein gp120, blocking CD4^+^-mediated viral entry into host cells (T cells, macrophages, and dendritic cells). Also, it has been shown that GRFT can inhibit HIV cell-to-cell transmission and the destruction of CD4^+^ T cells and block the transfer of HIV-1 to the lymph nodes, preventing CD4^+^ T cell infection, through binding to a protein on the surface of dendritic cells (DC-SIGN, i.e., dendritic cell intercellular adhesion molecule 3 (ICAM-3) grabbing non-integrin) by interactions between high-mannose glycans on the surface of the HIV gp120 and DC-SIGN [64,65,66,67].

These promising findings with GRFT led to the translation to clinical applicability of this molecule, namely through the development of a topical HIV pre-exposure prophylaxis (PrEP) gel, vehiculating GRFT in a carrageenan vaginal gel (PC-6500), which was applied in a Phase I clinical trial (NCT02875119) [68]. A 14-day randomized placebo-controlled, parallel-group, double-blind study was conducted in 13 HIV-seronegative, healthy, non-pregnant women aged 24–45 to evaluate the safety, pharmacokinetics, pharmacodynamics, and immunogenicity of PC-6500 applied once daily. The results of this trial were recently reported, showing that no significant adverse events were recorded in the laboratory and clinical results or histopathological assessments of the cervicovaginal mucosa, and no GRFT antibodies were detected in the serum. PC-6500 gel demonstrated no systemic absorption, as GRFT levels in blood remained below the lower limit of quantification (10 ng/mL) across the 14-day dosing period, and it remained active against HIV in the presence of vaginal fluids in vitro. Cervicovaginal proinflammatory responses and ectocervical transcriptome changes were not detected during the administration period. Decreased cytokine and chemokine levels in the cervicovaginal lavages indicate a possible anti-inflammatory effect of PC-6500 gel. As a further advantage, the PC-6500 gel also displayed anti-HPV and anti-HSV-2 activity, reducing HPV infection and HSV-2 infection/shedding, hence enhancing the anti-HIV effectiveness of this potential preventive medication [69].

The development of Q–Griffithsin (Q-GRFT), a genetically modified GRFT where a methionine residue is replaced by a glutamine residue, leads to a protein less susceptible to oxidation and therefore more stable [70]. The translation of this protein into clinical practice was achieved by the development of a Q-GRFT-based HIV PrEP topical enema, which has completed a Phase I clinical trial (NCT04032717). The trial consisted of a 28-day randomized, placebo-controlled, double-blind test, and it was conducted in 15 healthy adults, HIV-seronegative, aged 18–45 years, who practice receptive anal intercourse, to evaluate the safety and acceptability of the rectal application of Q-GRFT. Colorectal tissue samples were collected at several time points (baseline, 1 h, and 24 h) after a single administration to assess the effects of the Q-GRFT enema on the human rectal mucosa in vivo by evaluating a panel of epithelial junction proteins that address epithelial barrier integrity, whose stable expression displays a non-inflammatory environment, as well as CD4+ T cell distribution. The Q-GRFT enema did not change the expression or distribution of putative HIV susceptibility biomarkers regarding epithelial distribution, and there were no alterations in CD4^+^ T cells at any of the three time points, indicating that Q-GRFT is safe and acceptable [71,72].

Studies of GRFT and its derivatives continue, and recent advances include a highly productive recombinant system for GRFT point-of-care manufacturing within 24 h, using cell-free protein synthesis systems based on *Escherichia coli*, and another on plant extracts, using ALiCE^®^ (almost living cell-free expression) technology derived from tobacco BY-2 cells. Borhani et al. optimized gene sequences for improved protein expression, conducted small-scale reactions, and produced active, functional GRFT within 24 h. The study demonstrated that both systems could generate biologically active GRFT capable of antiviral activity against HIV-1 gp120 through an ELISA binding assay, but also against SARS-CoV-2, which was confirmed by an anti-SARS-CoV-2 pseudovirus inhibition test. The work highlights a scalable, portable, and rapid approach for decentralized manufacturing of antiviral biologics, which could be deployed at sites of emerging viral outbreaks for early intervention [73].

In a different approach, Steinbach-Rankins and Palmer incorporated GRFT and Q-GRFT into fiber formulations, with pre- and probiotics and nanoparticle systems, for the treatment of several microbial infections in the female reproductive tract, including HIV. Briefly, Steinbach-Rankins and Palmer filed a European patent on antimicrobial activity against multiple sexually transmitted infections (STIs), specifically HIV-1, HSV-2, *Chlamydia trachomatis*, *Trichomonas vaginalis,* and *Gardnerella vaginalis*, as well as the method of production of GRFT and Q-GRFT incorporation into electrospun fibers with inulin, fructo-, xylo-, and galactooligosaccharides, pectin, and beta-glucans (prebiotics), as well as *Lactobacillus acidophilus* and *Lactobacillus crispatus* (probiotics), and nanoparticles designed for sustained release. Regarding HIV infection, electrospun fibers and nanoparticles displayed HIV-1 inhibition in vitro when incubated with TZM-bl cells, engineered HeLa cells that express CD4, CCR5, and CXCR4 receptors, and a Tat-driven luciferase gene for HIV measurements of infection, for 1 h or 24 h prior to infection, with IC_50_ values below 50 ng/mL, similar to free GRFT. Fibers and nanoparticles provided sustained release of GRFT and Q-GRFT over extended periods (up to 90 days), maintaining anti-HIV-1 activity, which suggests potential for long-lasting prophylaxis in vivo. Cytotoxicity results in vaginal epithelial (VK2/E6E7), ectocervical (Ect1/E6E7), and endocervical (End1/E6E7) cell lines indicate a high cell viability (>90%) after exposure to fibers and nanoparticles for up to 72 h, emphasizing their safety for vaginal application. Additionally, both free Q-GRFT and encapsulated Q-GRFT were co-administered with antiretrovirals in free and encapsulated forms from different classes: tenofovir (nucleoside reverse transcriptase inhibitor), raltegravir (integrase inhibitor), and dapivirine (non-nucleoside reverse transcriptase inhibitor). Results showed a synergistic effect between Q-GRFT and antiretroviral drugs, with combination formulations having IC_50_ values that were at least six times lower than those of the isolated compounds. Co-administration of compounds with different modes of action can enhance the prophylactic effect and reduce the dose required to achieve effectiveness against HIV-1 infections. For the remaining STIs, the findings suggest that the delivery platforms, especially fibers incorporating GRFT, Q-GRFT, and pre- and probiotics, showed promise in preventing or reducing infections, with notable efficacy demonstrated against HSV-2 and potential applicability to other bacterial infections [74].

Another relevant protein with the potential to address HIV-1 infection is C-phycocyanin (C-PC), a phycobiliprotein isolated from the microalgae *Spirulina* sp., which displayed promising results in silico and in vitro. C-PC was found to be non-toxic to TZM-bl cells and PBMCs (peripheral blood mononuclear cells) at concentrations up to 0.5 mg/mL (4.5 μM) and exhibited a dose-dependent, broad-spectrum antiviral activity against HIV-1 clades A-D, with IC_50_ values ranging from 0.0817 mg/mL (0.74 μM) to 0.1740 mg/mL (1.58 μM). These findings point to C-PC’s capacity to inhibit diverse circulating HIV-1 subtypes. Mechanistic studies indicated that C-PC directly targets key HIV-1 enzymes. Molecular docking predicted stable, high-affinity interactions with reverse transcriptase and protease, occupying critical catalytic and substrate-binding residues. In vitro enzymatic assays corroborated these predictions, showing that C-PC suppressed the activity of both reverse transcriptase and protease in a dose-dependent manner, supporting its role as a multi-target antiviral protein. In addition, C-PC can also mitigate oxidative stress, which is a well-established contributor to HIV-1 pathogenesis. HIV-infected cells exhibited high levels of mitochondrial ROS, which were significantly reduced upon C-PC treatment, as detected by MitoSOX and DCF-DA fluorescent probes. This reduction in oxidative burden was accompanied by decreased caspase-3/7 activation and lower Annexin V/PI staining, indicating protection against virus-induced apoptotic pathways [75].

Harb and Chow evaluated the methanolic and aqueous extracts of beach-cast macroalgae, a biomass source often wasted, for their anti-HIV activity and verified that aqueous extracts displayed more promising results than the methanolic ones, with the best results being obtained for extracts of *Alsidium seaforthii*, *Osmundaria obtusiloba*, *Dictyopteris jolyana*, *Zonaria tournefortii*, and *Spyridia clavate*, reaching inhibition above 90%. These extracts are known to be rich in polyphenols, tannins, and polysaccharides, which might be responsible for this relevant bioactivity; however, no mechanistic studies have been reported so far [76].

### 5.2. Small Molecules

In the domain of small molecules, Boopathi et al. reported the identification of piperazine derivatives and other bioactive compounds from the red seaweed *Haloplegma duperreyi* with anti-HIV-1 activity [77]. Piperazine derivatives have been frequently associated with anti-HIV activity through proposed modes of action, such as disruption of viral replication or modulation of host–virus interactions [78]. The authors initially evaluated seven crude extracts prepared using solvents of increasing polarity (hexane, acetone, ethyl acetate, chloroform, methanol, ethanol, and water), where the methanolic extract exhibited the strongest antiviral effect, achieving 89.13% at 100 µg/mL, with an IC_50_ of 8.405 µg/mL and a therapeutic index of 23. Subsequent fractionation of the methanolic extract produced 12 fractions, of which the PF3 fraction showed the highest activity, displaying 90.67% inhibition at 100 µg/mL and an IC_50_ of 5.557 μg/mL, which was comparable to that of the reference antiretroviral AZT, along with an improved therapeutic index of 31. PF3 characterization by FTIR, NMR, and GC–MS revealed five major compounds: piperazine (25.69%), naphthalene (30.6%), 2,4-Di-tert-butylphenol (11.49%), and *E*-15-heptadecenal (5.02%) [77]. Further studies are required to evaluate if there is a synergistic action between the components or if the activity is due to a single compound.

Subramaniam et al. evaluated aqueous and methanolic extracts (at 100 µg/mL) of marine brown algae *Padina tetrastromatica* for their HIV-1 in vitro activity and immunomodulatory effects on PBMCs. Both extracts induced interleukin-10 (IL-10) and interferon-α production by PBMCs, indicating a promising immunostimulatory effect. In HIV-1-infected PBMCs, the extracts inhibited HIV-1 clades C and A by more than 90% and 50%, respectively. The extracts significantly reduced viral p24 antigen and also inhibited reverse transcriptase activity (>50%) [79].

These recent findings indicate that the chemical diversity in algae, which ranges from complex sulfated carbohydrates to proteins and small molecules, can open the door to new therapeutic solutions for addressing HIV/AIDS. Nevertheless, many studies are still focused on extract evaluation and characterization, and thorough phytochemical analysis and isolation of the chemical constituents might be required to identify hit compounds in the near future.

## 6. Relevant Symbiosis and Microorganisms as Sources of Anti-HIV Compounds

**Figure 5:**
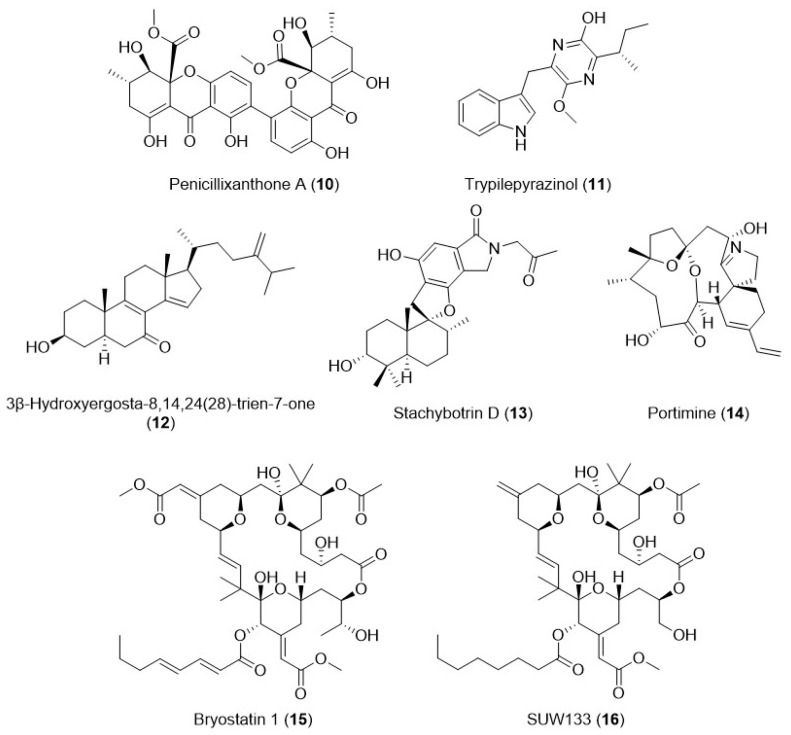
Examples of a marine natural product isolated from microorganisms or symbiosis (**10**–**15**) and a relevant synthetic bryolog (**16**) with promising anti-HIV activity.

Symbiosis is also a powerful source of chemical diversity in the ocean [80]. Octochorals, a group of cnidarians, contain host microorganisms that produce secondary metabolites with antiviral activities, exhibiting a wide range of chemical scaffolds, including terpenes, alkaloids, and peptides. Although many of these compounds have shown antibacterial, antifungal, and antiviral activities, the examples addressing anti-HIV activity are scarce. One example is penicillixanthone A (PXA, **10**, Figure 5), isolated from the fungus *Aspergillus fumigatus*, which often establishes symbiosis with jellyfish. This xanthone dimer, a complex natural chemical scaffold, showed potent anti-HIV-1 activity against CCR5-tropic HIV-1 SF162 and CXCR4-tropic HIV-1 NL4-3, with IC_50_ of 0.36 and 0.26 µM, respectively. Docking studies suggest the ability of this compound to bind to either CCR5 or CXCR4, preventing HIV entry into target cells [81].

The most studied marine fungi are of the genus *Penicillium*, which produces metabolites such as penicillin, griseofulvin, sorbicillacton A–B, isocoumarins, and sesquiterpenoids. These metabolites have shown a broad range of antiviral activities against important viruses, including HIV [82]. Trypilepyrazinol, a pyrazine derivative (**11**, Figure 5), and 3β-hydroxyergosta-8,14,24(28)-trien-7-one, an ergostane analog (**12**, Figure 5), were isolated from marine-derived fungus *Penicillium* sp. and displayed promising anti-HIV activity with IC_50_ values of 4.6 µM and 3.5 µM, respectively [83].

Stachybotrin D (**13**, Figure 5) is a phenylspirodrimane with antiviral activities, produced from the marine fungus *Stachybotrys chartarum* MXH-X73, isolated from the sponge *Xestospongia testudinaris*. This compound is a non-nucleoside reverse transcriptase inhibitor (NNRTI) of both wild-type HIV-1 (with an EC_50_ value of 8.4 μM) and five NNRTI-resistant strains (with EC_50_ values ranging from 0.7- to 2.8-fold the value obtained against the wild-type virus) [84].

Izumida et al. identified portimine (**14**, Figure 5), purified from the benthic marine dinoflagellate *Vulcanodinium rugosum*. This spirocyclic imine polyketide showed significant inhibition of HIV-1 replication at the nM range by directly targeting both the HIV-1 Gag and Pol proteins, as well as the reverse transcriptase. This makes this molecule a potent, multitarget lead compound to develop new anti-HIV-1 therapeutic options [85].

Divamides A and B are lanthipeptides produced by symbiotic bacteria living in the tunicate *Didemnum molle,* and their mechanism of action is hypothesized to occur through lipid binding, inhibiting viral entry into the cells [86].

*Bugula neritina*, also known as the brown bryozoan, is a marine animal that harbors an endobacterial symbiont, γ-proteobacterial *Candidatus Endobugula sertula*, responsible for the protection of *B. neritina* larvae from predation [87] and for the biosynthesis of bryostatins [88]. The first bryostatin structure characterized, bryostatin-1, was identified as a macrocyclic lactone (**15**, Figure 5) [89] and it is being extensively evaluated preclinically and clinically as a protein kinase C (PKC) agonist for cancer and Alzheimer’s disease [90,91]. Since the breakthrough of the importance of the PKC activation in the completion of the HIV replication cycle [92,93], researchers have been investigating bryostatin-1 as a potential LRA in HIV infection, contributing to the “shock and kill” strategy, and exploring whether bryostatin-1 plays additional roles that might aid in HIV eradication via a multitarget approach. Mehla et al. found that bryostatin-1 at 25 ng/mL (27.6 nM) inhibits HIV-1 infection against X4- and R5-tropic strains, independently of cellular receptors, and partially via a transient decrease in CD4/CXCR4 expression on CD4^+^-T cells. Bryostatin-1 at 25 ng/mL concurrently activates the PKC α isoform and, more significantly, the PKC δ isoform in monocytic and lymphocytic cells through the adenosine monophosphate (AMP)-activated protein kinase (AMPK) pathway, allowing the reactivation of HIV-1 latent viral infection without compromising CD4^+^-T cells [94]. Díaz et al. showed that bryostatin-1 at 100 ng/mL (110.4 nM) can also activate HIV-1 latent expression in human astrocytes, an HIV reservoir in the central nervous system, not only via PKC, but also by activating the transcriptional factor NF-ĸB, essential for the HIV-1 long terminal repeat (LTR) transcription, without impairing astrocyte viability [95]. López-Huertas et al. demonstrated that bryostatin-1 at 9 ng/mL (10 nM) and at 90 ng/mL (100 nM) reduced CCR5 expression on CD4^+^-T cells, antagonizing the anti-HIV effect of maraviroc, an antiretroviral used in clinical practice, which is only used for the treatment of CCR5-tropic HIV-1 infection [96]. Li et al. identified novel pathways for HIV latency reactivation in CD4^+^-T cells induced by bryostatin-1 at 45 ng/mL (50 nM), independent of PKC and NF-ĸB mechanisms through transcriptome analysis and target profiling. Results from the study suggest that the upregulation of purine and pyrimidine metabolism or cell-cycle arrest and apoptosis induction by upregulation of the p53 signaling pathway by bryostatin-1 reactivates HIV-latent CD4^+^-T cells [97]. Hany et al. investigated the effect of bryostatin-1 as an anti-HIV-1 agent in human monocyte-derived macrophages (MDMs), a cellular reservoir in HIV infection. Results showed that bryostatin-1, at 18 ng/mL (20 nM), had a 10-fold anti-HIV effect against R5-tropic strains in MDMs when compared to the treatment with tumor necrosis factor plus interferon (TNF at 20 ng/mL + IFN at 10 ng/mL) (used as a control), which is known to lessen HIV-1 infection. It also increased MDMs’ survival rate by approximately 5%, compared to HIV-1-infected MDMs. Bryostatin-1’s anti-HIV mechanism in MDMs primarily involves the downregulation of CD4 expression. Additionally, it reduces the formation of HIV-1 mature capsid and matrix proteins, which are essential for HIV RNA packaging, assembly, and maturation of viral particles. In contrast to the HIV-reactivating effect in CD4^+^-T cells, bryostatin-1 does not enhance viral production in chronically HIV-infected macrophages [98]. One of the latest findings concerning this multitarget-drug candidate is reported by Li and co-workers. They evaluated whether bryostatin-1 improves CD8^+^-T cell performance in the context of chronic HIV infection, as CD8^+^-T cells are required for controlling disease progression. In CD8^+^-T cell exhaustion models that simulate chronic HIV infection, bryostatin-1 at 9 ng/mL stimulated CD8^+^-T cell proliferation and functionality by reducing exhaustion-related phenotypes (e.g., programmed cell death protein 1); altering exhaustion-associated transcription factors expression by upregulation of T cell-specific DNA-binding protein TCF-1 and downregulation of thymocyte selection-associated high mobility group box protein TOX, and by inducting the expression of a mitogen-activated protein kinase, MAPK-11, and production of interferon-γ [99].

These studies, in a nutshell, showed that bryostatin-1 not only acts against HIV by reactivating latent viruses through several pathways in CD4^+^-T cells and astrocytes, acting as an LRA, but also by downregulating HIV entry receptors in CD4^+^-T cells and macrophages, suppressing infection and viral production in macrophages, and modulating CD8^+^-T cell proliferation and function.

Nevertheless, the translation from the bench to clinical practice remains a challenge even for this versatile molecule. Notwithstanding its demonstrated potential as a multi-action agent for HIV cure strategies, only a single pilot Phase I clinical trial has been conducted, revealing that bryostatin-1 did not elicit detectable activation of HIV RNA transcription in antiretroviral-suppressed patients, despite its safety and tolerability after a single-dose regimen of 10 or 20 μg/m^2^ [100]. This outcome may be attributed to the fact that the peak plasma concentration attained in these patients following a single administration was significantly below the nanomolar levels necessary for ex vivo HIV reactivation [101]. Another hurdle for the clinical translation of bryostatin-1 is its low natural abundance, as 14 tons of *B. neritina* only yielded 18 g of bryostatin-1 (0.00014%), and the environmental and economic burden associated with harvesting marine organisms [102]. Total synthesis of bryostatin 1 remains a challenge. Although Wender et al. unveiled a scalable synthesis of bryostatin-1, which reduced the initial 79 steps reported by Keck and co-workers [103] to 29 steps, providing 20 g of bryostatin-1, its synthesis remains complex, and the yield is low (4.8%) [104].

To overcome these obstacles and apply the knowledge gained about the anti-HIV mechanisms of action of bryostatin-1, bryostatin-1 analogs, also known as bryologs, were rationally designed to retain the same properties as bryostatin-1 while improving synthetic accessibility and tolerability. Marsden and co-workers characterized several bryologs in the context of HIV infection. They verified that these synthetic analogs, in CD4^+^-T cells, reduced the surface expression of HIV entry receptors CD4, CXCR4, and CCR5 and inhibited HIV replication in X4- and R5-tropic strains at 100 nM, and induced more latently infected CD4^+^-T cells than bryostatin-1 without a large increase in potentially damaging proinflammatory cytokines TNF-α, IL-2, and MIP-1α at 10 nM [105]. A particularly active analog, SUW133 (**16**, Figure 5), has provided in vivo proof of concept. In humanized BLT mice, a mouse model for investigating HIV persistence and pathogenesis on antiretroviral therapy, it induced the expression of latent HIV viruses and drove the death of a fraction of reactivated cells without compromising tolerability [106]. In more recent breakthroughs, a study showed that a single administration of allogeneic human peripheral blood natural killer cells with SUW133 delayed viral rebound after antiretroviral therapy interruption for a period of approximately 12 weeks and eliminated the viral reservoir from the splenocytes in a subset of humanized BLT mice infected with HIV-1 [107]. This compound also shows promising results in studies addressing synergistic effects with other bioactive molecules [108].

Overall, bryostatin-1 now functions primarily as a mechanistic template rather than a standalone drug candidate. In addition to PKC-centered approaches, it has established multi-pathway strategies for latency reversal, anti-HIV effects, and immune modulation. It inspired the development of safer and selective bryologs, as well as marine-based combination therapies.

Cyanobacteria have also been recognized as a prolific source of bioactive metabolites with antiviral properties, including anti-HIV [109,110,111,112,113]. Early work established that extracts from cultured cyanobacteria contain sulfonic acid–containing glycolipids (sulfoglycolipids) that inhibit HIV-1 replication in cell culture, reducing viral protein expression and syncytium formation, indicating interference with key steps in the viral lifecycle [114]. Lectins, such as cyanovirin-N isolated from *Nostoc ellipsosporum*, bind with high affinity to high-mannose oligosaccharides on the HIV envelope glycoprotein gp120, blocking attachment and fusion with host CD4^+^-T cells at nanomolar concentrations [115]. Similarly, scytovirin and microvirin, isolated from *Scytonema* and *Microcystis* species, respectively, exhibit strong neutralizing activity by targeting gp120 and preventing viral entry, with microvirin displaying favorable potency and lower cytotoxicity profiles in vitro [116,117]. *Oscillatoria agardhii* agglutinin homolog (OAAH) can also inhibit viral replication, syncytium formation, and virus uptake and translocation [118].

Further studies should focus on the in vivo safety, tolerability, and efficacy evaluation of marine-based combination therapies, providing a stronger foundation for clinical trials.

## 7. Conclusions

Table 1 summarizes the chemical families, mechanisms of action, and sources of the marine-derived compounds with the most promising anti-HIV activity described in this work.

Marine ecosystems remain a widely unexplored source of bioactive compounds. Habitat complexity and the biodiversity present in our oceans enable the development of valuable metabolites with intrinsic potential to interact with multiple pathogens, including HIV. From small molecules to complex polysaccharides and proteins, which often display multitargeted mechanisms of action, marine ecosystems continue to provide molecules with valuable therapeutic applications. Promising results concerning the translation from the ocean to the prophylaxis of HIV showcase how these products are safe and, therefore, promising agents to be included as active substances or adjuvants in drugs and medical devices. In multiple studies based on extract screening, the identification of possible hit compounds will bridge the gap between natural product chemistry and synthetic chemistry, reducing the need for extensive capture of often endangered species. The study of marine species, as well as their symbiotic associations, is therefore a relevant field in the discovery of novel anti-HIV agents.

## Figures and Tables

**Table 1:** Promising marine natural products, their chemical classes, sources, and mechanisms of action against HIV.

Natural Product	Chemical Class	Source	Mechanism of Action
Spongouridine (**1**)	Arabinosyl glycoside	*Tectitethya crypta*	Led to the development of AZT (**2**), a reverse transcriptase inhibitor and the first approved drug for HIV therapeutics
Ansellone J (**3**)	Sesterterpenoid	*Phorbas* sp.	HIV-1 latency reversal activity
Phorone C (**4**)	Sesterterpenoid	*Phorbas* sp.	HIV-1 latency reversal activity
2-Bromoalsidin (**5**)	Brominated pyrrolactam	*Stylissa massa*	Viral protein R inhibition
Mollamide F (**8**)	Thiazoline peptide	*Didemnum molle*	HIV integrase inhibition
Erythro-*N*-dodecanoyl-docosasphinga-(4*E*,8*E*)-dienine (**9**)	Fatty acid derivative	*Litophyton arboreum*	HIV-1 protease inhibition
Penicillixanthone A (**10**)	Xanthone dimer	*Aspergillus fumigatus*	Entry inhibition
Trypilepyrazinol (**11**)	Pyrazine derivative	*Penicillium* sp.	Unknown
3β-Hydroxyergosta-8,14,24(28)-trien-7-one (**12**)	Ergostane analog	*Penicillium* sp.	Unknown
Stachybotrin D (**13**)	Phenylspirodrimane derivative	*Stachybotrys chartarum*	Non-nucleoside reverse transcriptase inhibition
Portimine (**14**)	Polyketide	*Vulcanodinium rugosum*	Replication and reverse transcriptase inhibition
Bryostatin-1 (**15**)	Macrocyclic lactone derivative	*Candidatus Endobugula sertula*	Multitarget activity
Cnidarins (CNID 1–3)	Protein	*Synthecium* sp.	Entry/Fusion inhibition
Griffithin	Lectin protein	*Griffithsia* sp.	Entry inhibition
Divamides A and B	Lanthipeptides	Symbiotic bacteria living in tunicate *Didemnum molle*	Unknown
C-Phycocyanin	Phycobiliprotein	*Spirulina* sp.	Multitarget activity

## Data Availability

No new data were created or analyzed in this study. Data sharing is not applicable to this article.
